# Discordance of Non-HDL and Directly Measured LDL Cholesterol: Which Lipid Measure is Preferred When Calculated LDL Is Inaccurate?

**DOI:** 10.1155/2013/502948

**Published:** 2013-04-23

**Authors:** Lawrence Baruch, Valerie J. Chiong, Sanjay Agarwal, Bhanu Gupta

**Affiliations:** ^1^James J. Peters VA Medical Center, Bronx, NY 10468, USA; ^2^Mt. Sinai School of Medicine, NY, USA; ^3^Confluence Health, Moses Lake, WA 98837, USA; ^4^St. Luke's Hospital, University of Missouri, Kansas City, MO 64111, USA

## Abstract

*Objective*. To determine if non-HDL cholesterol (N-HDL) and directly measured LDL cholesterol (D-LDL) are clinically equivalent measurements. *Patients and Methods*. Eighty-one subjects recruited for 2 cholesterol treatment studies had at least 1 complete fasting lipid panel and D-LDL performed simultaneously; 64 had a second assessment after 4 to 6 weeks, resulting in 145 triads of C-LDL, D-LDL, and N-HDL. To directly compare N-HDL to D-LDL and C-LDL, we normalized the N-HDL by subtracting 30 from the N-HDL (N-HDL_A_). *Results*. There was significant correlation between N-HDL_A_, D-LDL, and C-LDL. Correlation was significantly greater between N-HDL_A_ and C-LDL than between N-HDL_A_ and D-LDL. A greater than 20 mg/dL difference between measures was observed more commonly between N-HDL_A_ and D-LDL, 29%, than between C-LDL and N-HDL_A_, 11% (*P* < 0.001), and C-LDL and D-LDL, 17% (*P* = 0.028). Clinical discordance was most common, and concordance was least common between N-HDL and D-LDL. *Conclusions*. Our findings suggest that N-HDL cholesterol and D-LDL cholesterol are not clinically equivalent and frequently discordant. As N-HDL may be superior to even C-LDL for predicting events in statin-treated patients, utilizing N-HDL to guide therapy would appear to be preferable to D-LDL when C-LDL is inaccurate.

## 1. Introduction

Calculated low density lipoprotein cholesterol (LDL) is the cornerstone of lipid lowering therapy [1–3]. In certain clinical situations, namely, the fed state or when triglycerides are greater than 400 mg/dL, calculated LDL is inaccurate and the guidelines recommend the use of directly measured LDL cholesterol [1].

We previously reported that calculated and directly measured LDL are not clinically equivalent when targeting ATP III goals with lipid lowering therapy in a significant number of patients [4]. Based on this finding, we recommended that non-HDL cholesterol (N-HDL), an accepted, inexpensive, and guideline-based measure of atherogenic particles, available from the routine lipid panel, replace directly measured LDL (D-LDL) when the calculated LDL (C-LDL) is inaccurate [4]. However, few studies are available comparing the clinical equivalence of calculated LDL, directly measured LDL, and non-HDL cholesterol measurements. Therefore, we evaluated the clinical equivalence of N-HDL and D-LDL which would allow us to determine which lipid measure is more appropriate when C-LDL is inaccurate.

## 2. Methods

Our population consisted of 81 subjects from the Bronx Veterans Affairs Medical Center recruited for 2 research studies from January 2007 through March 2009. The studies incorporated simultaneous measurement of direct LDL with a Siemens Advia Chemistry System and a complete fasting lipid panel at 2 time intervals, baseline, and after 4 to 6 weeks of therapy. C-LDL was calculated using the Friedwald formula: total cholesterol—HDL − triglycerides/5. Non-HDL cholesterol was calculated as total cholesterol—HDL cholesterol. The studies were approved by the institutional review board of the Bronx VA Medical Center and registered at http://clinicaltrials.gov/ (NCT00762164 and NCT00762229). All subjects signed an informed consent document. 

The NCEP ATP III guidelines, which established that N-HDL goals are 30 mg/dL above those for LDL (e.g., an individual with an LDL goal <100 has an N-HDL goal of <130), formed the basis for our comparisons between the measures of LDL (direct and calculated) and N-HDL [1, 3]. Thus, for assessment of placement in the same lipid goal cut points, LDL goals of <160, <130, <100, and <70 mg/dL corresponded to N-HDL goals of <190, <160, <130, and <100 mg/dL, respectively [1, 3]. To “normalize” N-HDL to LDL values for direct numerical comparisons, 30 was subtracted from N-HDL (e.g., an N-HDL of 155 was “normalized” to a value of 125, 155 − 30 = 125) to create the “adjusted” N-HDL (N-HDL_A_). 

Correlation between the lipid parameters was determined using Pearson's correlation coefficient. 

The relationship between C-LDL, D-LDL, and N-HDL was also evaluated from a clinical perspective based on the following rationale. In clinical practice, adjustment of lipid lowering therapy is primarily based on the LDL numerical value (e.g., 112 mg/dL) and the ATP III goal (e.g., LDL < 100 mg/dL, N-HDL < 130 mg/dL in a patient with diabetes) [1, 3]. Furthermore, doubling the dose of any statin results in an additional 6% reduction in LDL [5]. Finally, minor changes in LDL are not generally considered to be clinically meaningful. As such, when comparing any 2 of the lipid values (e.g., N-HDL to D-LDL, N-HDL to C-LDL) they were considered “*clinically concordant*” when fulfilling the following criteria: (1) placement in the same ATP III goal cut point range (e.g., 100–129 when comparing C-LDL and D-LDL, an N-HDL of 100–129 and a C-LDL 70–99) [1, 3], (2) <6% difference between the values (e.g., C-LDL versus N-HDL_A_, and C-LDL versus D-LDL) which represents the incremental LDL lowering provided by a single titration of statin dose (e.g., from simvastatin 20 to 40 mg [5]), and (3) <10 mg/dL difference between the 2 values (a difference that clinicians may consider meaningful, thus not concordant, even when the values being compared are in the same ATP III cut point and differ by <6%, e.g., when D-LDL is 181 mg/dL and N-HDL_A_ is 171 mg/dL). 

We considered the numerical values “*clinically discordant*” when they fulfilled the following criteria: (1) placement in different ATP III goal cut point ranges [1, 3], (2) ≥12% difference between the 2 values (representing the incremental LDL lowering provided by two statin titration steps, e.g., from atorvastatin 20 to 80 mg [5]), and (3) ≥10 mg/dL difference between the values (as clinicians may *not* consider the values discordant, even when they are in different ATP III cut points and differ by ≥12% when the values being compared differ by <10 mg/dL, e.g., when N-HDL_A_ is 66 mg/dL and D-LDL is 75 mg/dL).

Categorical data were compared using descriptive statistics from Minitab 15th Edition (State College, PA). One sample *t*-test and analysis of variance (ANOVA) were used to assess within-group and between-group differences in lipid values. A *P* value < 0.05 was considered statistically significant for these analyses. Differences in correlation coefficients were evaluated by Fisher's *z*-transformation. A *z*-value ≥ 2.0 was considered significant for these analyses. Microsoft Excel 2007 and SPSS, version 19.0 (SPSS Inc., an IBM company), were used to analyze study data.

Our sample size was limited by the number of subjects who enrolled in each of the respective protocols.

## 3. Results

A total of 81 subjects had at least one lipid triad of C-LDL, D-LDL, and N-HDL performed simultaneously. Of these 81 subjects, 64 successfully completed their respective studies and had lipids assessed at baseline and followup. Sixteen subjects had lipids assessed only at baseline, and 1 patient only had a followup, as his baseline lipid assessment was non-fasting. This resulted in a total of 145 total triads of C-LDL, D-LDL, and N-HDL that were included in the analysis. 

Baseline characteristics are shown in Table 1. Subjects tended to be elderly, overweight, hypertensive, and predominantly male. At baseline, N-HDL_A_ was less than direct and calculated LDL in 81% and 68% of subjects, respectively; D-LDL was greater than C-LDL in 66% of patients. 

**Figure 1:**
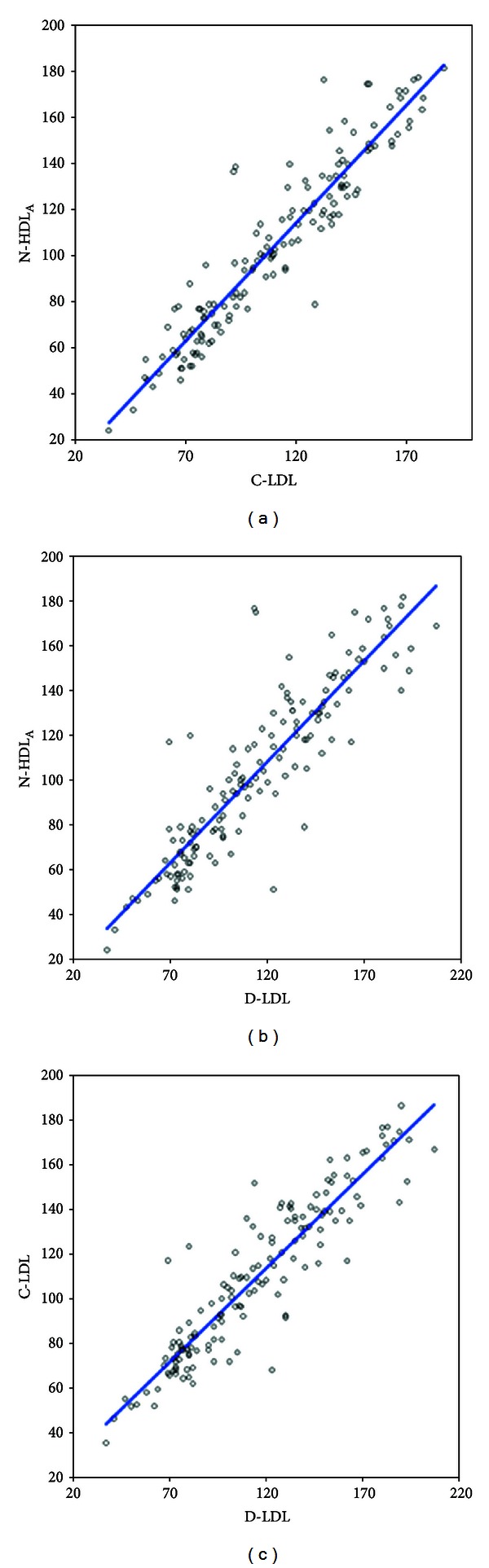
Regression plots for D-LDL-C, C-LDL, and N-HDL_A_ for all values (*n* = 145). (a) N-HDL_A_ with C-LDL (*r* ^2^ = 0.89). (b) N-HDL_A_ with D-LDL (*r* ^2^ = 0.80). (c) D-LDL with C-LDL (*r* ^2^ = 0.86).

There was significant correlation between N-HDL_A_, D-LDL, and C-LDL. For the entire cohort, which included all 145 baseline and follow-up values, the correlations for N-HDL_A_ with C-LDL, *r* ^2^ of 0.89 (Figure 1(a)), N-HDL_A_ with D-LDL, *r* ^2^ of 0.80 (Figure 1(b)), and C-LDL with D-LDL, *r* ^2^ of 0.86 (Figure 1(c)) were strong. The strength of the correlation between N-HDL_A_ and C-LDL was statistically significantly greater than between N-HDL_A_ and D-LDL for both the baseline (*z* = 2.02) and entire cohorts (*z* = 2.66). 

**Figure 2:**
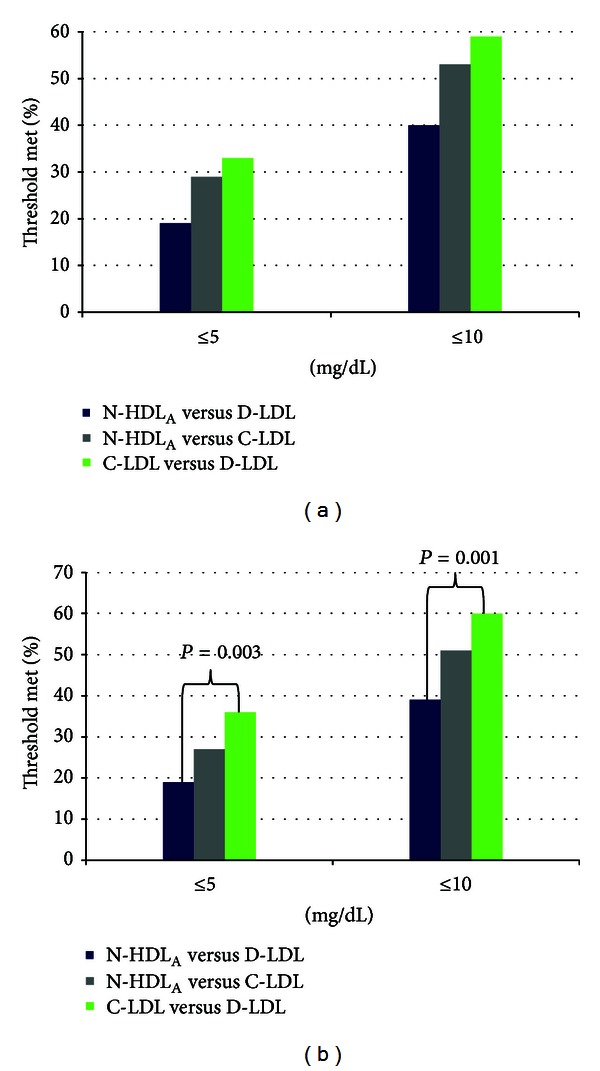
Percentage of patients whose values are near each other from a numerical perspective, <5 and <10 mg/dL. (a) Baseline values (*n* = 80), approached statistical significance for the between group differences (*P* = 0.054). (b) All values in the study (*n* = 145).

At baseline, *clinical concordance* (as defined in Section 2) between D-LDL and N-HDL was present in only 20% of patients, as compared to 36% between C-LDL and N-HDL and 34% between C-LDL and D-LDL. This difference approached statistical significance when comparing D-LDL and N-HDL to C-LDL (*P* = 0.064). Furthermore, when analyzed from a numerical perspective of clinical equivalence, values within 5 or 10 mg/dL of each other, this was least commonly observed with N-HDL_A_ and D-LDL in the baseline and entire cohort (Figure 2). A statistically significant difference was observed between N-HDL_A_ versus D-LDL and C-LDL versus D-LDL for both 5 and 10 mg/dL for the entire cohort (Figure 2(b)). 

*Clinical discordance* (as defined in Section 2) between non-HDL and the 2 measures of LDL was noted on at least 1 occasion in 47% of patients with respect to D-LDL and in 37% with respect to C-LDL, while C-LDL and D-LDL were discordant in only 30% of patients (*P* = 0.075). A similar pattern emerged when looking at only the baseline lipid values, with discordance noted between N-HDL and D-LDL in 31% of patients, as compared to 23% between N-HDL and C-LDL and 15% between direct and calculated LDL (*P* = 0.039 versus N-HDL and D-LDL). 

**Figure 3:**
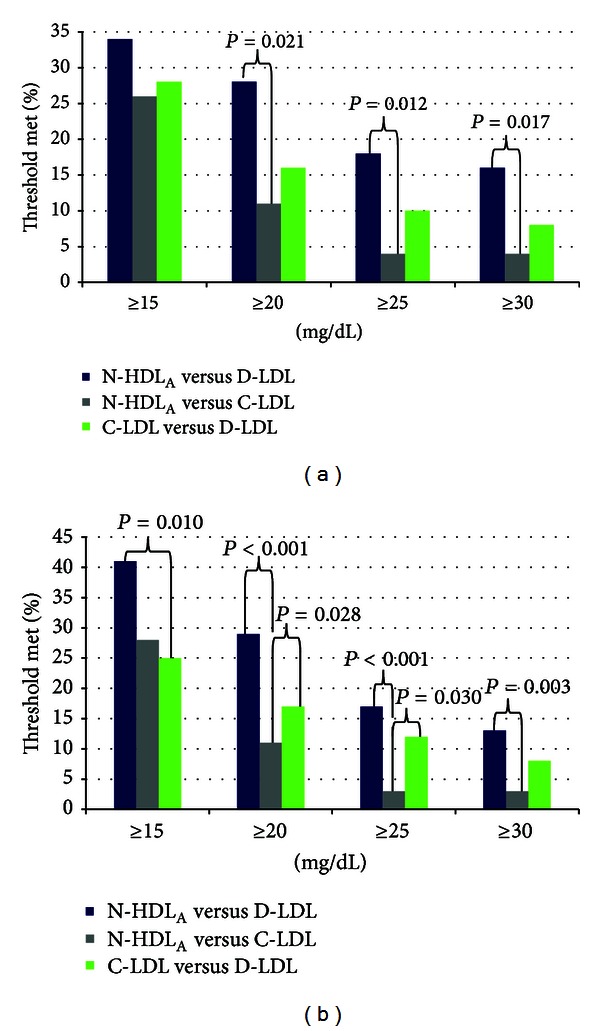
Percentage of patients with a difference between N-HDL_A_, D-LDL and C-LDL of 15, 20, 25, and 30 mg/dL. (a) Baseline values (*n* = 80). (b) All values in the study (*n* = 145).

Moreover, a clinically meaningful percentage of patients had a greater than 20 mg/dL difference between the various measures across the dataset, for both baseline and all values (Figure 3). This was statistically significantly more common when analyzing all values, between N-HDL_A_ and D-LDL, 29%, when compared to C-LDL and N-HDL_A_, 11% (*P* < 0.001) or C-LDL and D-LDL, 17% (*P* = 0.028) (Figure 3(b)). Similar statistically significant and clinically meaningful findings were observed when analyzing only the baseline values (Figure 3(a)). A number of patients manifested even larger differences between the lipid measures, differences that clinicians would consider highly meaningful. This was observed most frequently when comparing N-HDL_A_ and D-LDL, and was statistically significant for a number of values and lipid comparisons (Figure 3). 

**Figure 4:**
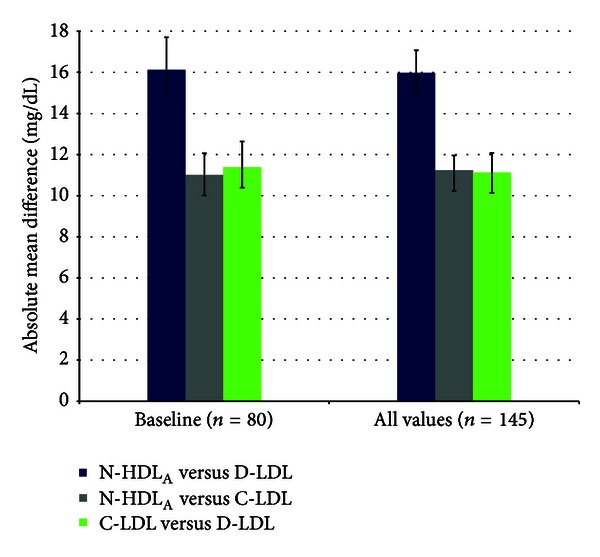
Mean absolute difference with standard deviation between N-HDL_A_, D-LDL, and C-LDL for baseline values (*n* = 80) and all values (*n* = 145) in the study. The difference and standard deviation are the largest between N-HDL_A_ and D-LDL in both cohorts. Between-group differences were statistically significant, with N-HDL_A_ and D-LDL significantly different from both of the other lipid measurements for the baseline and entire cohorts, *P* = 0.010 and <0.001, respectively.

Similarly, the mean absolute difference between the various lipid measures was the greatest between D-LDL and N-HDL_A_, approximately 16 mg/dL, as compared to approximately 11 mg/dL for N-HDL_A_ to C-LDL and D-LDL to C-LDL (Figure 4). 

## 4. Discussion

Our analysis demonstrates a linear correlation between “adjusted” N-HDL and measures of LDL used in clinical practice, calculated and direct LDL. Despite this correlation, clinical discordance was observed in more than one in three patients, and it was more common for N-HDL to be discordant than concordant with either C-LDL or D-LDL. It is evident that N-HDL does not consistently provide similar clinical information to either measures of LDL in individual patients, a finding parallel to what we had seen previously when comparing C-LDL to D-LDL. This dissociation between clinical concordance and correlation results from the correlation coefficient being a summary statistic, rather than a statistic that defines relationships in individual patients. 

Moreover, N-HDL and D-LDL, the recommended alternative lipid measures when C-LDL is inaccurate [1, 3] appear to be the most divergent of the three lipid “pairs,” with N-HDL_A_ generally lowest, D-LDL highest, and C-LDL intermediate. Similarly, the average difference between lipid “pairs” was the greatest between N-HDL_A_ and D-LDL. N-HDL_A_ and D-LDL had the fewest number of patients whose values were very close to each other, within 5 mg/dL, and the greatest number with highly discrepant values (e.g., >20 mg/dL). Furthermore, N-HDL_A_ and D-LDL had the fewest concordant and most discordant values. Consistent with these findings, the weakest correlation was between N-HDL and D-LDL.

Consequently, when C-LDL is inaccurate, recommending measurement of either N-HDL or D-LDL appears questionable, as these 2 lipid measures may not reliably provide the same clinical information in individual patients. Adopting such an approach will result in different treatment decisions, resulting in less intensive therapy when the primary target is N-HDL, and more intensive therapy, even with respect to C-LDL, when D-LDL is the primary target. As there are fewer extreme differences from a numerical perspective between N-HDL and C-LDL, and N-HDL affords the additional advantages of the absence of additional cost and potential superiority to even C-LDL in predicting cardiovascular risk [6–11], the use of N-HDL seems preferable in situations where C-LDL is inaccurate. The use of non-HDL in this setting is consistent with the growing body of support for non-HDL as a target of therapy [6–11], with some even advocating that it replaces *calculated* LDL as the primary target of therapy [12].

### 4.1. Study Limitations

Our study is limited by the use of a single commercial assay, in a modest number of a select, male, veteran population from a single center. Criteria for clinical concordance and discordance were based on our understanding of what clinicians would consider clinically meaningful. In addition, our patients are not representative of those in whom assessment of D-LDL is recommended, as all were fasting and had triglyceride levels of less than 400 mg/dL. Despite these limitations, our study remains relevant for a number of reasons. Clinicians routinely use commercially available D-LDL assays, while our criteria for clinical concordance and discordance consisted of 3 criteria, any 1 of which clinicians may consider clinically meaningful. Moreover, N-HDL becomes more valuable as a predictor of risk as the triglycerides increase and non-HDL-C represents a secondary target of therapy when triglycerides are greater than 200 mg/dL [1].

## 5. Conclusion

N-HDL and D-LDL are the most divergent of the various lipid “pairs”, on opposite “poles” of C-LDL, and thus do not appear to be clinically equivalent. The current findings strengthen our previous recommendation that N-HDL, and not D-LDL, be used in clinical situations where C-LDL is inaccurate, as N-HDL is a validated target of therapy, free of additional cost, and incorporated into existing guidelines. The clinical discordance between all 3 lipid measures, N-HDL, D-LDL, and C-LDL, raises the issue of whether any of these lipid measures is the best predictor of cardiovascular risk and begs the question of whether alternative lipid measures, such as apoB or LDL particle number, should become the primary target of therapy [13–16]. Prospective research is needed to compare all these lipid measures to identify the best predictor of cardiovascular risk.

## Figures and Tables

**Table 1:** Baseline characteristics.

Variable	Initiation (*n* = 81)
Age (years)	60
Weight (kg)*	92
Height (cm)*	176
Male	38 (100%)
Hypertension	25 (66%)
Diabetes mellitus	9 (24%)
Low density lipoprotein (mg/dL)—calculated*	141
Low density lipoprotein (mg/dL)—direct*	149
Total cholesterol (mg/dL)	216
HDL (mg/dL)	49
Non-HDL (mg/dL)	160
“Adjusted” non-HDL (mg/dL)	130
Triglycerides (mg/dL)	130

*One patient had nonfasting lipids; his baseline LDL measurements, triglycerides, HDL, and “adjusted” non-HDL were therefore not included. Two patients did not have an assessment of height and weight.
